# Hair Growth-Combing through the Various Determinants

**DOI:** 10.4103/0974-7753.77529

**Published:** 2010

**Authors:** Dilip Gude

**Affiliations:** Department of Internal Medicine, AMC, 3^rd^ Floor, Medwin Hospital, Chirag Ali Lane, Nampally, Hyderabad, Andhra Pradesh-500 001, India

Sir,

The mechanisms governing hair growth have been elusive despite tremendous research in the field. There is a plethora of causative factors implicated. An autosomal recessive form of alopecia was mapped to the chromosome 13q14.11–13q21.33 and the role of homozygous truncating mutations in P2RY5 (member of a subgroup of Oleoyl-L-α-lysophosphatidic acid receptors) which encode an orphan G protein–coupled receptor.[1] Cytoplasmic calcium and reactive oxygen species have been shown to regulate, and integrate monomeric G-proteins, actin cytoskeleton, protein kinases and phospholipid-modulating enzymes thereby facilitating the spatial localization of growing tip. Increased expression of thymosin β-4 in the hair follicles has been shown to promote hair regrowth in transgenic mice. Human scalp hair follicles (HFs) are both a source and a target of thyrotropin receptor hormone (TRH) as HFs express not only TRH receptors (TRH-R) but also TRH itself at the gene and protein level. TRH operates as a potent hair-growth stimulator via mechanisms of hair-shaft elongation, prolongation of the hair cycle growth phase (anagen), antagonism of its termination by TGF-β2 and increasing the proliferation and inhibition off apoptosis of hair matrix keratinocytes. Some of these effects may be mediated by reducing the ataxia telangiectasia-mediated (ATM)/Atr (ATM and Rad3 related)-dependent phosphorylation of p53.[2] It has also been shown that the physiological concentrations of the thyroid hormones T3 and T4 enhance the KERATIN 15 promoter activity. They also stimulate the expression of the immuno-inhibitory surface molecule CD200, induce apoptosis and differentiation and inhibit clonal growth of these progenitor cells *in vitro*. Further studies are warranted to discern the endocrine controls of human epithelial cells.

The frontal bald area of patients with androgenetic alopecia has lower proliferation rate that results in follicular miniaturization. There is increased DNA damage beyond the capacity of cells to repair in advanced cases as evidenced by significantly higher levels of X-ray cross complementing-1 (XRCC1) and p53 expression and significantly lower expression of proliferating cell nuclear antigen and apurinic/apyramidinic endonuclease 1. The damaged cells undergo an alternative pathway in being eliminated through apoptosis.[3] Isoflavone has been shown to promote hair growth-associated with melanogenesis by increasing calcitonin gene-related peptide production in the sensory neurons which in turn leads to increase in insulin-like growth factor-1 production in the hair follicle. α-methylspermidine is known to induce hair growth characterized by pigmentation, growing hair follicles, proliferation of follicular keratinocytes and upregulation of β-catenin.[4] The protein WNT and its inhibitor Dickkopf protein (DKK) are believed to be primary determinants of hair follicle spacing as transgenic DKK overexpression has been shown to reduce overall epithelial appendage density. Neural Wiskott-Aldrich syndrome protein (N-WASP) has a link with WNT and is a positive regulator of β-catenin-dependent transcription and differentiation and cycling of HF progenitor cells.[5]

There are a variety of newer therapeutic approaches targeting these determinants in rejuvenating the hair growth. We have come a long way understanding the biology of hair growth and the continuing research shall definitely help us disentangle the mysterious knots of hair growth.
